# Ecological causes of morphological evolution in the three-spined stickleback

**DOI:** 10.1002/ece3.581

**Published:** 2013-05-06

**Authors:** Rowena Spence, Robert J Wootton, Iain Barber, Mirosław Przybylski, Carl Smith

**Affiliations:** 1 School of Biology, University of St. AndrewsSt. Andrews, KY16 8LB, UK; 2 Institute of Biological, Environmental and Rural Sciences, Aberystwyth UniversityAberystwyth, Ceredigion, SY23 3DA, UK; 3 Department of Biology, University of LeicesterLeicester, LE1 7RH, UK; 4 Department of Ecology and Vertebrate Zoology, University of ŁódźŁódź, Poland

**Keywords:** Adaptation, calcium concentration, *Gasterosteus aculeatus*, natural selection, nuptial coloration, phenotypic adaptation, selective predation

## Abstract

The central assumption of evolutionary theory is that natural selection drives the adaptation of populations to local environmental conditions, resulting in the evolution of adaptive phenotypes. The three-spined stickleback (*Gasterosteus aculeatus*) displays remarkable phenotypic variation, offering an unusually tractable model for understanding the ecological mechanisms underpinning adaptive evolutionary change. Using populations on North Uist, Scotland we investigated the role of predation pressure and calcium limitation on the adaptive evolution of stickleback morphology and behavior. Dissolved calcium was a significant predictor of plate and spine morph, while predator abundance was not. Stickleback latency to emerge from a refuge varied with morph, with populations with highly reduced plates and spines and high predation risk less bold. Our findings support strong directional selection in three-spined stickleback evolution, driven by multiple selective agents.

## Introduction

The phenotypic adaptation of populations through natural selection in response to local environmental conditions is a cornerstone of evolutionary theory (Thompson 1917; Maynard Smith 1958). Despite its significance, our understanding of the ecological mechanisms that underpin adaptive evolutionary change is remarkably poor (Schluter 2001; MacColl 2011). One approach to identifying causes of selection is to match spatial variation in phenotype with putative agents of selection in the environment. Understanding the extent of genetic and phenotypic divergence, either as a response to geographic variation or from constraints imposed by gene flow and stabilizing selection, is considered a key question in evolutionary biology (Schluter 2001; Coyne and Orr 2004; Benkman and Parchman 2013).

The three-spined stickleback (*Gasterosteus aculeatus* L.) shows considerable morphological, physiological, and behavioral variation across its range and is an unusually tractable vertebrate model for understanding the mechanism of phenotypic adaptations in relation to local environmental conditions (Wootton 1984, 2009; Bell et al. 1993; Peichel et al. 2001; Schluter et al. 2010). It is a small teleost fish with a wide distribution across the temperate and boreal Northern Hemisphere, occurring in marine, anadromous, and freshwater populations, the latter occupying a diversity of aquatic habitats (Reimchen 1994; Wootton 2009).

Sticklebacks lack the typical scales of most fishes, possessing instead bony lateral plates, dorsal spines, and a spined pelvic girdle (Wootton 1976). The variation in the extent of these bony elements among populations is substantial. Three morphs have been described on the basis of the number and arrangement of these bony plates: (1) the *complete* morph has a continuous row of plates from immediately behind the head to the caudal peduncle; (2) the *partial* morph has an anterior row of plates, then a length of the body that lacks plates, succeeded by a posterior row of plates reaching to the tail; (3) the *low* morph has only an anterior row of plates and the remainder of the body is naked (Hagen and Gilbertson 1973; Wootton 1976). Within a population, there is variation in the number and size of plates within each morph, but the three morphs can be defined unambiguously. While marine and anadromous forms tend to display *complete* or *partial* morphs, the majority of freshwater populations belong to the *low* morph, and a few populations show extreme reduction of plates, pelvic girdle, and dorsal spines (Moodie and Reimchen 1976; Giles 1983; Bell et al. 1985, 1993). This morphological variation can be understood in terms of the species' evolutionary history (Bell 2009; Wootton 2009; Schluter et al. 2010). The marine form, which is phenotypically and genetically more uniform, is ancestral, while freshwater populations derive from repeated invasions of freshwater habitats by anadromous ancestors (Bell 1976; Withler and McPhail 1985). The plate and spine reduction that accompanies the colonization of freshwater predominantly reflects genetic change (Münzing 1959; Lindsey 1962; Hagen and Gilbertson 1973), though modest phenotypic plasticity has been recognized (Frommen et al. 2011). Genetic evidence suggests that the same alleles (*Eda* in the case of lateral plates and *Pitx1* in the case of pelvic structures) underlie the independent evolution of plate and spine reduction over a wide geographic range (Colosimo et al. 2004, 2005; Cresko et al. 2004; Shapiro et al. 2004; Coyle et al. 2007; Chan et al. 2010). However, while the genetic basis to armor reduction is well described, a less tractable problem has been to understand the selective agents that favor the evolution of different plate and spine morphs in contrasting environments. Several hypotheses have been proposed (Myhre and Klepaker 2009; Wootton 2009; Schluter et al. 2010), though the two most enduring are the nature and intensity of predation (Hagen and Gilbertson 1973; Reimchen 1980; Bell et al. 1993), and the bioavailability of dissolved calcium (Giles 1983; Spence et al. 2012).

Bony plates, in combination with pelvic and dorsal spines, function in defense against predators (Hoogland et al. 1957; Reimchen 1983, 1994). Several studies show nonrandom predation on sticklebacks (Bańbura et al. 1989), and sticklebacks with robust plates and spines are more likely to survive attacks by predatory vertebrates (Reimchen 1992, 2000). However, there may be a trade-off between number of lateral plates and swimming ability, resulting in selection for reduced body armor in the absence of predation pressure (Taylor and McPhail 1986; Reimchen 2000; Barrett et al. 2008). Among *low* morph populations, there is an association between predation pressure and spine length and the number of lateral plates (Reimchen 1994; Bell 2001). However, in the virtual absence of predators, a rapid evolutionary transition from the *complete* to the *low* morph is still observed (Le Rouzic et al. 2011). Among populations, lateral plate reduction is the most frequently encountered variable armor trait, followed by reduction in spine length. The complete loss of lateral plates and pelvic girdle reduction/loss is rare (Klepaker and Østbye 2008). Reimchen (1980) suggested that in the absence of predatory fishes, pelvic structures and spines may make sticklebacks more vulnerable to aquatic insect predators, such as dragonfly nymphs, which use these appendages to grasp sticklebacks. Plateless fish may additionally benefit from an enhanced fast-start escape response for evading predators, particularly those with low pursuit efficiency, such as birds and invertebrates (Bergstrom 2002). However, compelling evidence for this hypothesis is scarce and indirect (Marchinko 2009; Zeller et al. 2012).

An alternative explanation for variation in plate and spine reduction among freshwater populations was offered by Giles (1983) who noted that in North Uist in the Scottish Outer Hebrides, plateless and spine-deficient sticklebacks occur only in waters with particularly low concentrations of dissolved calcium. Thus the extreme reduction in bony elements in these populations may result from calcium limitation. Calcium is a vital component of the diet of vertebrates and is required for the normal development and maintenance of the skeletal system, with calcium deficiency resulting in well-characterized growth deformities (Nordin 1960). Experimentally depriving juvenile three-spined sticklebacks of calcium had the effect of limiting their growth, particularly for morphs with the most extensive spine and plate development (Spence et al. 2012), suggesting calcium availability may play a role in the evolution of stickleback plate and spine morph, at least under conditions in which calcium is limiting. Other occurrences of plateless and spine-deficient sticklebacks, in the Queen Charlotte Islands, British Columbia (Moodie and Reimchen 1976) and Cook Inlet, Alaska (Bourgeois et al. 1994), are also restricted to habitats with low concentrations of calcium. Bell et al. (1993) pointed out that the two hypotheses, predation pressure and calcium availability, are not necessarily mutually exclusive, as predatory fishes will themselves be calcium limited and are likely to be less abundant in low calcium waters. However, in North Uist predatory fish, principally brown trout (*Salmo trutta*), regularly occur in the same waters as plateless and spine-deficient sticklebacks (Giles 1983; Campbell 1985).

The three-spined stickleback populations of North Uist offer an exceptional opportunity to correlate spatial variation of phenotype with putative agents of selection in the environment. The aim of this study was to investigate the role of predation pressure and calcium limitation on stickleback adaptive evolution. North Uist supports numerous populations of both *low* plated and spine, plate and pelvic girdle reduced or deficient sticklebacks in freshwater lochs that vary in both predation pressure and dissolved calcium concentration. The west coast of North Uist is characterized by a band of calcium-rich shell-sand grassland, termed the *machair* that supports rich vegetation and alkaline, biologically productive lochs. In the central and eastern regions the *machair* gives way to blanket peat bogs with acidic and oligotrophic lochs (Beveridge 2001; Friend 2012). Calcium concentrations in freshwater lochs on North Uist reported by Giles (1983) ranged from 28.3 to 0.94 mg/L, with a striking gradient of declining dissolved calcium from West to East. The chief fish predator of sticklebacks on North Uist is the brown trout, which occurs naturally in the majority of lochs. The only other freshwater fishes to occur are eels (*Anguilla anguilla*), which occur infrequently (two elvers caught in 4 years of sampling), nine-spined sticklebacks (*Pungitius pungitius*), and small, localized populations of arctic charr (*Salvelinus alpinus*). In sea lochs and freshwater lochs with open marine connections, migratory salmon (*Salar salar*) and sea trout (*S. trutta*) also occur, though these were excluded from the present study. Fishing rights on North Uist are controlled by the North Uist Estate, which maintains detailed fish catch records spanning several decades.

We predicted that in the case that predation is the major selective agent for stickleback morphology, stickleback plate and spine morph would be dependent on trout abundance, with sticklebacks displaying greater plate and spine development at sites with high predation risk. Fish behavior is also sensitive to predation risk (Brown and Braithwaite 2004). Thus as an additional test of the effect of predation, we predicted that population differences in the “latency to emerge from a refuge” of sticklebacks, estimated as the time taken for individual fish to emerge from shelter in a novel environment sensu Brown and Braithwaite (2004), would also correlate with trout abundance, with sticklebacks from populations exposed to high predation risk showing longer emergence times than those from low predation risk populations.

If calcium availability is the major agent of selection for stickleback plate and spine morph evolution, we predicted morphology to vary as a function of dissolved calcium concentration, with sticklebacks displaying the most limited plate and spine development at sites where dissolved calcium concentrations were lowest. Changes in plate and spine morphology are relatively rapid, with a reversal in morph dominance possible in as little as 10 years (Klepaker 1993; Bell et al. 2004). Thus for dissolved calcium concentration to drive plate and spine morph evolution, we also predicted that current dissolved calcium concentrations in North Uist lochs would match estimates made 30 years previously by Giles (1983).

## Materials and Methods

### Stickleback morphology

We collected sticklebacks and water samples from 34 freshwater lochs on North Uist (20 sites in 2010, 11 in 2011, three in 2012), and compared stickleback plate and spine morphology, and dissolved calcium concentrations among lochs. These lochs included all 27 lochs originally sampled by Giles (1983) in 1980, with seven additional sites. All sampled lochs were isolated and without populations of migratory sticklebacks. Sticklebacks were collected using long-handled dip nets, killed with anesthetic (benzocaine) and fixed in 4% buffered formalin. Samples comprised a minimum of 30 individuals and were restricted to adult fish >26 mm Standard Length (SL, measured from the tip of the snout to the origin of the tail) to ensure morphological characteristics were fully developed (Bell 1981). After fixing, fish were transferred to 70% ethanol for 24 h, stained with 0.08% alizarin red in 1% KOH for 24 h, rinsed in water for 24 h, and stored in 70% ethanol. Plate and spine morphs were scored as one of four possible morphs: *low,* with only thoracic plates, but with and dorsal and pelvic spines; *plateless,* with no lateral plates but no reduction in the spines or pelvic girdle; *spine-deficient plated* with thoracic plates, but no dorsal and pelvic spines; *spine-deficient plateless,* with no lateral plates and reduced or absent dorsal spines, ventral spines, and pelvic girdle (Campbell 1985). To maximize sample sizes for analysis, *plateless, spine-deficient plated,* and *spine-deficient plateless* morphs are referred to collectively as *minimal* morph sticklebacks. The *complete* morph, with a row of lateral plates along the length of the body including a caudal keel, and robust dorsal and pelvic spines, and the *partial* morph, with an unplated region separating the thoracic and caudal plates, and dorsal and pelvic spines, which are associated with marine and estuarine populations, were not encountered.

Water samples were collected in 1 L sample bottles from below the water surface in at least 1 m depth. Dissolved calcium concentration was measured by titration using a LaMotte calcium hardness kit (LaMotte Company, Chestertown, MD) in accordance with the manufacturers instructions. Predator abundance was estimated on the basis of brown trout catches. Lochs were scored on an ordinal scale of 1–4 based on historical catch records over a 30-year period, with one indicating lochs with the lowest historical brown trout catches and 4 the highest. Lochs were scored independently by the North Uist Estate manager, who is responsible for collating all trout catch returns. While relatively crude, this measure of predator abundance provided a robust index of predation risk for sticklebacks among populations, derived from comprehensive long-term catch records for simple ecological systems, with brown trout the only abundant piscivorous fish in freshwater lochs. Anecdotal evidence and catch return data indicate consistent brown trout abundances among sampling sites over several decades (G. Macdonald, pers. comm.). Catch records were based on trout caught by fly-fishing, as this is the sole fishing technique permitted on North Uist. “Fly” fishing is a misnomer, as the lures used by fly fisherman more typically imitate small fish than invertebrates, and the favored fly patterns used on North Uist are fish mimics. Thus, trout catches effectively provide an index of attack rates on small fish, of which sticklebacks are the primary representatives.

### Stickleback behavior

To assay stickleback latency to emerge from a refuge, live fish were collected from 16 lochs on North Uist, 12 in 2011, and a further four in 2012, with 8 *low* morph and 8 *minimal* morph populations, four each from lochs with high and low predation abundance (Table 1). A subset of lochs was used, rather than all 34 sites, due to logistical constraints and to provide a balanced design. Fish were collected by sweeping long-handled dip nets through littoral vegetation and kept outdoors in aerated 60 L tubs containing water and aquatic plants taken from the loch from which they were captured. This method of fish collection appeared indiscriminate with respect to fish size, coloration, or boldness. Fish were fed once daily with a mixture of frozen bloodworm and zooplankton.

**Table 1:** Lochs on North Uist sampled in 1980 and 2010–12 with estimates of dissolved calcium concentration (mg/L), stickleback morph (see text for definitions), and rank predator abundance (1 = low abundance, 4 = high)

		1980		2010–12		
				
Site	OS grid reference	Dissolved calcium (mg/L)	Morph	Dissolved calcium (mg/L)	Morph	Rank trout abundance
Loch Fada	875 705	2.25	*spine-deficient plateless*	3.06	*spine-deficient plateless*	4
Loch á Bharpa^1^	837 664	1.90	*spine-deficient plateless*	1.42	*spine-deficient plateless*	1
Loch nan Eun^1^	845 675	2.50	*spine-deficient plateless*	3.83	*spine-deficient plateless*	4
Loch Huna^1^	813 665	1.65	*spine-deficient plateless*	1.87	*spine-deficient plateless*	4
North Sgadabhagh	868 685	1.40	*spine-deficient plateless*	1.00	*spine-deficient plateless*	3
Loch na Maighdein^1^	893 683	0.94	*spine-deficient plateless*	1.49	*spine-deficient plateless*	2
Loch na Moracha^1^	846 665	2.50	*spine-deficient plateless*	1.14	*spine-deficient plateless*	4
Loch á Bhuird^1^	883 675	2.20	*spine-deficient plateless*	2.18	*spine-deficient plateless*	4
Loch nan Geadh^1^	888 706	1.80	*spine-deficient plateless*	1.87	*spine-deficient plateless*	2
Loch Tormasad	820 650	2.50	*plateless*	2.90	*plateless*	3
Loch Bhereagbhat	882 723	0.94	*plateless*	1.80	*plateless*	2
Loch Bruist	775 683	1.80	*plateless*	2.82	*fish absent*	–
Loch an Daimh^1^	889 678	3.55	*low*	3.24	*low*	1
Loch nan Geireann^1^	845 725	1.80	*low*	1.87	*low*	2
Loch Croghearraidh	716 712	20.70	*low*	10.78	*low*	2
Loch Sgaraigh^1^	717 705	16.90	*low*	13.49	*low*	2
Loch Sanndaraigh^1^	735 685	23.50	*low*	11.19	*low*	*2*
Loch Steineabhat	875 743	2.80	*low*	3.95	*low*	3
Loch na Morgha^1^	870 743	4.70	*low*	3.50	*low*	3
Loch Hosta^1^	727 727	28.30	*low*	13.72	*low*	4
Loch Dubhasairidh	772 673	3.30	*low*	3.38	*low*	2
Loch nan Athan^1^	778 668	9.40	*low*	4.06	*low*	3
Loch á Charra	779 689	4.50	*low*	3.31	*low*	2
Loch nan Strùban	808 647	4.00	*low*	3.27	*plateless*	4
Loch an Toim	794 658	3.30	*low*	1.63	*plateless*	4
Loch na Creige	883 737	3.70	*low*	3.55	*low*	2
Bogach Maari^1^	860 719	–	*–*	1.01	*spine-deficient plateless*	1
Loch Eubhal^1^	725 713	–	*–*	12.57	*low*	3
South Sgadabhagh	878 665	–	*–*	2.5	*spine-deficient plateless*	3
Loch nan Cethir Eilean	858 664	–	*–*	3.2	*spine-deficient plated*	2
Loch Hungabhat	873 724	–	*–*	2.92	*low*	3
Wee Fada	793 667	–	*–*	3.26	*plateless*	1
Loch Deòireabhat	892 662	–	*–*	2.35	*spine-deficient plateless*	3
Loch Eisiadair	807 728	–	*–*	4.21	*low*	2

Data for 1980 from Giles (1983), and 2010–12 the present study.

1 Populations used in boldness assay.

Assays of latency to emerge from a refuge were conducted within 24 h of fish capture and followed the protocol of Brown and Braithwaite (2004). Assays were conducted in glass aquaria measuring 300 (length) × 200 (width) × 200 (depth) mm. The aquarium had a gravel substrate and water to depth of 150 mm. Each population was assayed in water from the loch from which it originated to mimic the water chemistry to which they were naturally exposed. Test aquaria were illuminated from above with a 20 W lamp with a daylight bulb to standardize lighting conditions. Given the small volume of the test aquaria and standardized illumination, light transmittance during trials was comparable for all experimental fish, irrespective of the degree to which loch water was peat stained. The aquarium was bisected with a removable clear plastic screen placed 100 mm from one end. Aquatic plants that reached the water surface were placed behind the screen to provide a refuge area of dense submerged vegetation. The remaining 20 mm section of the rest of the aquarium was bare of plants. Fish were tested individually, the test fish being selected randomly from stock aquaria and gently released into the vegetated end of the test aquarium. After a 5 min acclimation period the plastic screen was raised allowing the fish to explore the whole aquarium. An observer recorded the time taken for the fish to emerge a full body length from the vegetated end into the open part of the aquarium. A fish that failed to emerge within 10 min was assigned a score of 600 sec (a total of 18 fish of 144 tested were assigned this truncated score). After completion of a trial, the test fish was recaught and its SL measured. A total of 12 young of the year fish from each of the 16 populations were tested, in a random order, with no fish tested more than once. Mean SL (±SD) of fish used in behavioral assays was 30.1 (±7.2) mm, range 26–35 mm.

### Data analysis

The direction of change in dissolved calcium concentrations of the 26 North Uist lochs sampled by Giles (1983) in 1980 and by us in 2010–12 was compared using a paired test. A logistic regression analysis was conducted to predict stickleback plate morph for the 33 populations from which three-spined sticklebacks were collected using dissolved calcium concentration and brown trout abundance as predictors. For this analysis, stickleback populations, which were monotypic, were classed as either *low* morph or *minimal* morph. *Minimal* morph populations comprised *plateless, spine-deficient plated* and *spine-deficient plateless* morphs categorized as a single group. For the behavioral study, a two-factor analysis of covariance (ANCOVA) was used to test for the effects of plate morph and predation regime on the time taken to emerge from a refuge, with fish SL included as a covariate and population as a block factor. A Fligner-Killeen test was used to measure homoscedasticity. Diagnostic plots of residuals against fitted values, and a normal QQ plot of residuals were used to assess assumptions of normality. Data for time to emerge from cover were log_10_ transformed for analysis.

## Results

### Stickleback morphology

We detected no change in plate and spine morphs over the 30-year interval since Giles' (1983) sampling in 1980, with the exception of two sites: Lochs nan Strùban and an Toim (Table 1). In these lochs fish had previously been categorized by Giles (1983) as *low* morph, while we found fish to be *plateless* in both cases. In one site, Loch Bruist, in which Giles (1983) identified a *plateless* population, we failed to capture any three-spined sticklebacks. In Loch Dubhasairidh, a single individual of the *partial* morph was collected, with all remaining fish being *low*. There was no significant change in dissolved calcium concentration in 26 lochs between 1980 and 2010–12 (Wilcoxon signed rank test: *Z* = 1.54, *n* = 26, *P* = 0.124; Table 1). The mean (±SD) dissolved calcium concentration for the 26 sites reported by Giles (1983) was 5.88 (±7.54) mg/L and 3.93 (±3.52) mg/L in the present study.

**Figure 1:**
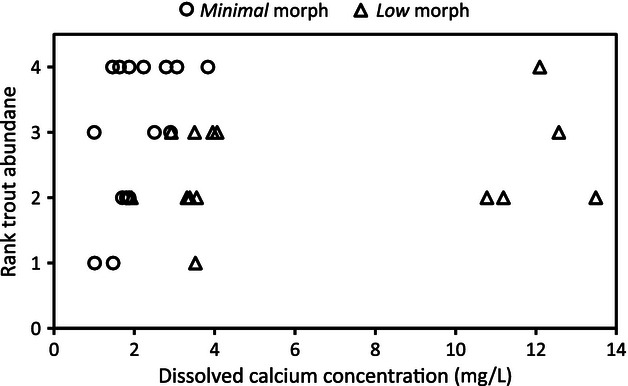
The relationship between three-spined stickleback morph, dissolved calcium concentration (mg/L), and brown trout abundance for 33 lake populations on North Uist (Scotland). Circles denote *minimal* morph, triangles *low* morph three-spined sticklebacks.

Logistic regression analysis showed a significant effect of dissolved calcium concentration on stickleback plate morph, but no significant effect of trout abundance (Fig. 1). A test of the full model against a constant only model was statistically significant, indicating that the predictors reliably distinguished between *low* and *minimal* morphs (*χ*^2^_df:2_ = 24.42, *P* < 0.001). Nagelkerke's *R*^2^ = 0.699 indicated a moderately strong relationship between prediction and grouping. Prediction success overall was 85% (87% for *low* morph and 83% for *minimal*). Wald's criterion demonstrated that only dissolved calcium concentration made a significant contribution to prediction (Wald = 7.55, *P* = 0.006). Trout abundance was not a significant predictor of stickleback morph (Wald = 1.99, *P* = 0.159). The interaction of calcium and predation was not significant (*P* > 0.1) and, therefore, was not included in the model.

### Stickleback behavior

**Figure 2:**
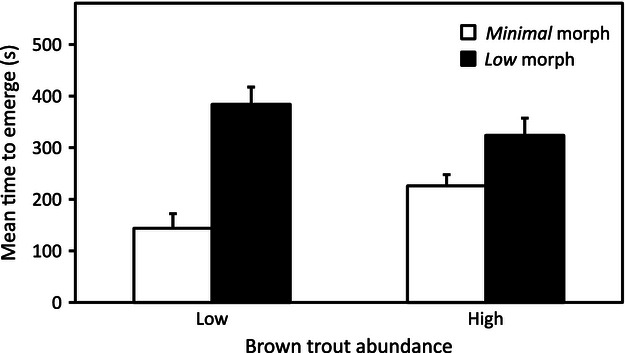
Mean (±SE) time taken to emerge (s) from vegetation by *minimal* and *low* morph three-spined sticklebacks from North Uist (Scotland) lakes with low and high brown trout abundance.

The interaction between morph and predator abundance on the time taken by fish to emerge from a refuge was not significant (*F*_1, 75_ = 3.99, *P* = 0.071), and there was no main effect of predator abundance (*F*_1, 75_ = 0.19, *P* = 0.297). However, there was a significant effect of morph (two-way ANCOVA, *F*_1, 11_ = 12.54, *P* = 0.005), with *minimal* morph fish emerging faster than *low* morph (Fig. 2). Notably, *minimal* morph fish from populations with low predator abundance emerged significantly faster than those from high predator abundance lochs (one-way ANCOVA, *F*_1,5_ = 9.49, *P* = 0.027), while there was no significant effect of predator abundance on emergence times of *low* morph fish (one-way ANCOVA, *F*_1,5_ = 0.07, *P* = 0.803) (Fig. 2). Fish SL was not a significant covariate in the analysis (full model, *F*_1,7_ = 3.32, *P* = 0.096), but there was a significant difference in fish SL at the population level (*F*_1,175_ = 6.82, *P* = 0.010) and so it was retained in the model.

## Discussion

Adaptive phenotypic change in response to a changed environment is a fundamental concept in evolutionary biology, and a number of influential examples of rapid radiations, including Darwin's finches (Grant and Grant 2006), Caribbean anoles (Losos 1992), and Hawaiian silverswords (Baldwin and Sanderson 1998), have been described. However, despite its significance, the identification of the mechanisms, both genetic and ecological, that underpin adaptive phenotypic change are often incompletely understood. In the present study, we investigated spatial variation in the phenotype of three-spined sticklebacks on the Hebridean Island of North Uist, and examined the role of dissolved calcium concentration and predation risk as agents of selection. Dissolved calcium concentration was a significant predictor of plate and spine morph, while predator abundance was not. Stickleback latency to emerge from a refuge varied with plate morph, with *minimal* morph fish sensitive to predator abundance at the population level.

*Minimal* morph sticklebacks were typically found in water with calcium concentrations <3 mg/L, the exception (also noted by Giles 1983) was Loch Geireann where fish were the *low* morph despite a calcium concentration of <2 mg/L (Table 1). Dissolved calcium concentrations measured in the present study failed to show a directional change in comparison with those collected by Giles (1983), implying stability in water quality and stickleback morph over several decades (Table 1) and suggesting that any selection imposed by calcium availability would have been consistent over this period. It was notable that the greatest changes in mean calcium concentration between the two periods resulted largely from changes in the four lochs with the highest dissolved calcium concentrations: Lochs Croghearraidh, Sanndaraigh, Hosta, nan Athan), possibly indicating imprecision in the measurement of dissolved calcium concentrations by Giles (1983) or in the present study.

A possible explanation for the persistence of the *low* morph at a calcium concentration <2 mg/L may simply be because the necessary mutations to produce a *minimal* morph may not yet have arisen or been introduced into the Loch Geireann population. The stickleback populations in Loch an Toim and Loch nan Strùban were the only ones in which plate morph had changed since 1980, from *low* to *plateless*, with calcium concentrations in both lochs having fallen below 3 mg/L in the intervening period. Notably the pattern of stickleback morphs showed the same pattern of distribution as the striking habitat gradient that is a feature of North Uist (Beveridge 2001; Friend 2012), with the low morph associated with the *machair* in the West and the *minimal* morphs corresponding with low calcium acidic lochs in the central and eastern regions (Table 1).

We found no evidence for a direct relationship between stickleback morph and predation risk. This finding contradicts those of Bell et al. (1993), Reimchen (1994), and Baker et al. (2010) who reported plate reduction only in lakes with no native predators, though it is consistent with the finding of MacColl and Chapman (2011) and Zeller et al. (2012). Predatory brown trout occurred in all the lochs we sampled, and risk of predation was relatively high in several lochs where stickleback populations expressed *minimal* morphs (e.g., Loch nan Eun, Loch na Moracha, and Loch á Bhuird). Our measure of predation risk was indirect; using trout catches over an extended period as a surrogate for predation risk. However, angler's catches are likely to provide a more robust assessment of predation risk than sampling trout abundance, as catch rates provide a measure of attack rates on small fishes, which abundance estimates fail to do. Thus, we are confident that our estimates of predation risk were representative of selection from predators among populations.

Although we did not quantify avian or mammal (Eurasian otter, *Lutra lutra*) predation rates, there is no reason to expect the pressure imposed by these highly mobile predators to vary systematically between lochs, and brown trout likely represent the most significant predatory threat to sticklebacks. Otters also rarely include sticklebacks in their diet (Watt 1995), while avian predators, such as gray herons (*Ardea cinerea*), have limited abundance throughout the Outer Hebrides (Marquiss 1989). Black-throated (*Gavia arctica*) and red-throated divers (*G. stellata*) are summer visitors to freshwater lochs in the Outer Hebrides, though at low densities and there is seldom more than a single pair on a loch where they do occur (Gibbons et al. 1997). Common (*Sterna hirundo*) and Arctic terns (*S. paradisaea*) are also summer visitors, and are known to feed on sticklebacks (Wootton 1984), but are not as abundant in the Western Isles as further south, and are associated with feeding on marine rather than freshwater fish species (Buxton 1985; Clode and Macdonald 2002). The potential role on invertebrate predators in shaping stickleback plate and spine morph was proposed by Reimchen (1980), who argued that the presence of plates and spines in sticklebacks might make them more susceptible to invertebrate predators, which use these features to grasp sticklebacks during predatory attacks. Consequently reduced plates and spines are predicted where these predators are abundant. Predatory odonate larvae were encountered during sampling in North Uist, and though they were not systematically surveyed, they were encountered more frequently in the calcium-rich *machair* lochs. This observation corresponds with the finding that odonate larval survival rates correlate with pH (Bell 1971). Thus these invertebrate predators were most frequently associated with the *low* rather than *minimal* stickleback morphs, which is the opposite outcome to that predicted if these predators were acting as selective agents on plate and spine morph. A study by Zeller et al. (2012) of a population of three-spined sticklebacks exposed to predation by fish and dragonfly larvae showed significant directional selection for elevated growth rates. Selection on plate morph was inconsistent, with both *low* and *complete* plate morphs showing greater survival in response to predator-induced selection.

A difficulty with our conclusions is the presence of some populations with no pelvic reduction even at extremely low concentrations of calcium, such as Loch nan Geireann in the present study, and in some Norwegian lakes (Klepaker 1993). Conversely, sticklebacks with pelvic reduction are found in Paxton Lake, British Columbia, which has a relatively high dissolved calcium concentration (Larson 1976). Further, sticklebacks in both Paxton Lake and four Norwegian lakes had a bimodal distribution of body armor within the same lake (Klepaker and Østbye 2008). Thus, other selective forces, such as buoyancy or osmoregulation, may be involved in determining plate morph and pelvic structures that have yet to be identified.

Adaptive variation in antipredator behavior in relation to predation risk is well documented in sticklebacks (Huntingford 1982; Giles and Huntingford 1984; Huntingford et al. 1994; Dingemanse et al. 2009). Fish from populations exposed to high predation risk are often more vigilant and display enhanced fright reactions. These differences appear to arise from an interaction between inherited and learned traits (Tulley and Huntingford 1987; Messler et al. 2007). In addition, exposure to predators can affect the heritability of specific behavioral traits, such as levels of activity and shoaling (Dingemanse et al. 2009). We detected a significant difference between stickleback morphs in antipredator behavior. Latency to emerge from a refuge among *minimal* morph populations varied significantly with predator abundance, while among better armored *low* morph fish it did not (Fig. 2). This result suggests that populations with reduced protection were especially sensitive to predation, possibly because they are more susceptible to predators. However, our finding that *low* morph fish were more cautious overall than *minimal* morph was unexpected. A possible explanation could be the difference in water clarity between the typical habitats of the respective morphs, with *low* populations found in clear, alkaline water where fish are more conspicuous, while *minimal* morph populations inhabit stained peaty water. The only other study of which we are aware linking stickleback morph with predation regime and antipredator behavior did not demonstrate a significant effect, though the study failed to replicate at the population level (Grand 2000). Behavioral differences among populations of the related nine-spined stickleback have also been attributed to predator pressure (Herczeg et al. 2009, 2012). The interaction between morph and predator abundance in the present study was not statistically significant, but was sufficiently low to warrant further investigation of a possible divergent response of different morphs to predation.

A further conclusion from this result is that although *minimal* morph sticklebacks experience selection pressure in response to predation, environmental constraints limit the evolution of morphological traits that would confer better antipredator protection. Both morphological and behavioral data indicate that there is a threshold calcium concentration below which lateral plates and pelvic spines cannot evolve, irrespective of predation pressure. Bell et al. (1993) observed that the frequency of pelvic reduction in lakes with introduced predatory species was increased at calcium concentrations <12 mg/L. Our data suggest that where native predators are present, the threshold is <3 mg/L. This threshold would also explain why there were no polymorphic populations detected in North Uist, but rather a bimodal distribution of populations of *low* and *minimal* morphs. A central role of calcium in driving morphological selection is probably limited to exceptionally calcium poor environments, as adequate quantities of calcium can usually be obtained by sticklebacks in their diet or from the surrounding water (Spence et al. 2012).

In conclusion, our study implicates dissolved calcium concentration as the principal agent of selection for stickleback plate and spine morph on North Uist, while predation selected against latency to emerge from a refuge in *minimal* morph populations. These results suggest *minimal* morph sticklebacks may be more susceptible to predation than better armored forms, but are constrained in the evolution of armor by calcium availability. These findings implicate more than one selective agent as the mechanism for phenotypic adaptation to local environmental conditions in the three-spined stickleback. While our study design did not permit us to measure the strength of selection of different variables it was clear that, at least where it is limiting, the bioavailability of calcium played a key role in the evolution of different plate and spine morphs, while fish behavior appeared more sensitive to predation risk. Overall our findings support strong directional selection in three-spined stickleback evolution, driven by multiple selective agents. Further insights into the causes of evolution using the stickleback, and other systems, can come through experimental manipulation of the putative agents of selection, combined with measurements of the strength of selection on adaptive traits (Schluter 2001; MacColl 2011).

## References

[b1] Baker JA, Wunda MA, Chock RY, Ackein L, Elsemore R, Foster SA (2010). Predation history and vulnerability: conservation of the stickleback adaptive radiation. Biol. Conserv.

[b2] Baldwin BG, Sanderson MJ (1998). Age and rate of diversification of the Hawaiian silversword alliance. Proc. Natl. Acad. Sci. USA.

[b3] Bańbura J, Przybylski M, Frankiewicz P (1989). Selective predation of the pike *Esox lucius*: comparison of lateral plates and some metric features of the three-spined stickle back *Gasterosteus aculeatus*. Zool. Scr.

[b4] Barrett RD, Rogers SM, Schluter D (2008). Natural selection on a major armor gene in threespine stickleback. Science.

[b5] Bell HL (1971). Effect of low pH on the survival and emergence of aquatic insects. Water Res.

[b6] Bell MA (1976). Evolution of phenotypic diversity in *Gasterosteus aculeatus* superspecies on the Pacific coast of North America. Syst. Zool.

[b7] Bell MA (1981). Lateral plate polymorphism and ontogeny of the complete morph of threespine sticklebacks (*Gasterosteus aculeatus*. Evolution.

[b8] Bell MA (2001). Lateral plate evolution in the threespine stickleback: getting nowhere fast. Genetica.

[b9] Bell MA (2009). Implications of a fossil stickleback assemblage for Darwinian gradualism. J. Fish Biol.

[b10] Bell MA, Francis RC, Havens AC (1985). Pelvic reduction and its directional asymmetry in threespine sticklebacks from the Cook Inlet Region, Alaska. Copeia.

[b11] Bell MA, Ortí G, Walker JA, Koenings JP (1993). Evolution of pelvic reduction in threespine stickleback fish: a test of competing hypotheses. Evolution.

[b12] Bell MA, Aguirre WE, Buck NJ (2004). Twelve years of contemporary armor evolution in a threespine stickleback population. Evolution.

[b13] Benkman CW, Parchman TL (2013). When directional selection reduces geographic variation in traits mediating species interactions. Ecol. Evol.

[b14] Bergstrom CA (2002). Fast-start swimming performance and reduction in lateral plate number in threespine stickleback. Can. J. Zool.

[b15] Beveridge E (2001). North Uist.

[b16] Bourgeois JF, Blouw DM, Koenings JP, Bell MA (1994). Multivariate analysis of geographic covariance between phenotypes and environments in the threespine stickleback, *Gasterosteus aculeatus*, from the Cook Inlet area, Alaska. Can. J. Zool.

[b17] Brown C, Braithwaite VA (2004). Size matters: a test of boldness in eight populations of the poeciliid *Brachyraphis episcopi*. Anim. Behav.

[b18] Buxton NE (1985). The current status and distribution of terns in the Outer Hebrides. Scott. Birds.

[b19] Campbell NR (1985). Morphological variation in the three-spined stickleback (*Gasterosteus aculeatus*) in Scotland. Behavior.

[b20] Chan YF, Marks ME, Jones FC, Shapiro G, Villarreal MD, Brady SD (2010). Adaptive evolution of pelvic reduction in sticklebacks by recurrent deletion of a Pitx1 enhancer. Science.

[b21] Clode D, Macdonald DW (2002). Invasive predators and the conservation of island birds: the case of American mink *Mustela vison* and terns *Sterna* spp. in the Western Isles, Scotland. Bird Study.

[b22] Colosimo PF, Peichel CL, Nereng K, Blackman BK, Shapiro MD, Schluter D (2004). The genetic architecture of parallel armor plate reduction in threespine sticklebacks. PLoS Biol.

[b23] Colosimo PF, Hosemann KE, Balabhardra S, Dickson G, Villarreal M, Grimwood J (2005). Widespread parallel evolution in sticklebacks by repeated fixation of Ectodysplasin alleles. Science.

[b24] Coyle SM, Huntingford FA, Peichel CL (2007). Parallel evolution of Pitx1 underlies pelvic reduction in Scottish threespine stickleback (*Gasterosteus aculeatus*. J. Hered.

[b25] Coyne JA, Orr HA (2004). Speciation.

[b26] Cresko WA, Amores A, Wilson C, Murphy J, Currey M, Phillips P (2004). Parallel genetic basis for repeated evolution of armor loss in Alaskan threespine stickleback populations. Proc. Natl. Acad. Sci. USA.

[b27] Dingemanse NJ, Wright F, Van der Plas J, Réale D, Schrama M, Roff DA (2009). Individual experience and evolutionary history of predation affect expression of heritable variation in fish personality and morphology. Proc. Biol. Sci.

[b28] Friend P (2012). Scotland.

[b29] Frommen JG, Herder F, Engqvist L, Mehlis M, Bakker TCM, Schwarzer J (2011). Costly plastic morphological responses to predator specific odour cues in three-spined sticklebacks (*Gasterosteus aculeatus*. Evol. Ecol.

[b30] Gibbons DW, Bainbridge IP, Mudge GP, Tharme AP, Ellis PM (1997). The status and distribution of the red-throated diver *Gavia stellata* in Britain in 1994. Bird Study.

[b31] Giles N (1983). The possible role of environmental calcium levels during the evolution of phenotypic diversity in Outer Hebridian populations of the three-spined stickleback, *Gasterosteus aculeatus*. J. Zool.

[b32] Giles N, Huntingford FA (1984). Predation risk and inter-population variation in antipredator behavior in the three-spined stickleback, *Gasterosteus aculeatus* L. Anim. Behav.

[b33] Grand T (2000). Risk-taking by threespine stickleback (*Gasterosteus aculeatus*) pelvic phenotypes: does morphology predict behavior?. Behavior.

[b34] Grant PR, Grant BR (2006). Evolution of character displacement in Darwin's finches. Science.

[b35] Hagen DW, Gilbertson LG (1973). The genetics of the plate morphs in freshwater three-spine sticklebacks. Heredity.

[b36] Herczeg G, Gonda A, Merila J (2009). Predation mediated population divergence in complex behavior of nine-spined stickleback (*Pungitius pungitius*. J. Evol. Biol.

[b37] Herczeg G, Gonda A, Kuparinen A, Merila J (2012). Contrasting growth strategies of pond versus marine populations of nine-spined stickleback (*Pungitius pungitius*): a combined effect of predation and competition?. Evol. Ecol.

[b38] Hoogland RD, Morris D, Tinbergen N (1957). The spines of sticklebacks (*Gasterosteus* and *Pygosteus*) as a means of defence against predators (*Perca* and *Esox*. Behavior.

[b39] Huntingford FA (1982). Do inter- and intraspecific aggression vary in relation to predation pressure in sticklebacks?. Anim. Behav.

[b40] Huntingford FA, Wright PJ, Tierney JF, Bell MA, Foster SA (1994). Adaptive variation in anti-predator behavior in the threespine stickleback. The evolutionary biology of the threespine stickleback.

[b41] Klepaker T (1993). Postglacial evolution in lateral plate morphs in Norwegian freshwater populations of the threespine stickleback (Gasterosteus aculeatus).

[b42] Klepaker T, Østbye K (2008). Pelvic anti-predator armour reduction in Norwegian populations of the threespine stickleback: a rare phenomenon with adaptive implications?. J. Zool.

[b43] Larson GL (1976). Social behavior and feeding ability in two phenotypes of *Gasterosteus aculeatus* in relation to their spatial and trophic segregation in a temperate lake. Can. J. Zool.

[b44] Le Rouzic A, Østbye K, Klepaker TO, Hansen TF, Bernatchez L, Schluter D (2011). Strong and consistent natural selection associated with armor reduction in sticklebacks. Mol. Ecol.

[b45] Lindsey CC (1962). Experimental study of meristic variation in a population of threespine sticklebacks, *Gasterosteus aculeatus*. Can. J. Zool.

[b46] Losos JB (1992). The evolution of convergent structure in Caribbean *Anolis* communities. Syst. Biol.

[b47] MacColl ADC (2011). The ecological causes of evolution. Trends Ecol. Evol.

[b48] MacColl ADC, Chapman SM (2011). A benthic predatory fish does not cause selection on armour traits in three-spined stickleback *Gasterosteus aculeatus* (Gasterosteiformes: Gasterosteidae). Biol. J. Linn. Soc.

[b49] Marchinko KB (2009). Predation's role in repeated phenotypic and genetic divergence of armor in threespine stickleback. Evolution.

[b50] Marquiss M (1989). Grey Herons *Ardea cinerea* breeding in Scotland: numbers, distribution, and census techniques. Bird Study.

[b51] Maynard Smith J (1958). The theory of evolution.

[b52] Messler A, Wund MA, Baker JA, Foster SA (2007). The effects of relaxed and reversed selection by predators on the antipredator behavior of the threespine stickleback, *Gasterosteus aculeatus*. Ethology.

[b53] Moodie GEE, Reimchen TE (1976). Phenetic variation and habitat differences in *Gasterosteus* populations of the Queen Charlotte Islands. Syst. Zool.

[b54] Münzing J (1959). Biologie, variabilitat und genetik von *Gasterosteus aculeatus* L. (Pisces) Untersuchungen im Elbegebiet. Int. Rev. Gesamt. Hydrobiol.

[b55] Myhre F, Klepaker T (2009). Body armour and lateral-plate reduction in freshwater three- spined stickleback *Gasterosteus aculeatus*: adaptations to a different buoyancy regime?. J. Fish Biol.

[b56] Nordin BEC (1960). Osteomalacia, osteoporosis and calcium deficiency. Clin. Orthop.

[b57] Peichel CM, Nereng KS, Ohgl KA, Cole BEL, Colosimo PF, Buerkle CA (2001). The genetic architecture of divergence between threespine stickleback species. Nature.

[b58] Reimchen TE (1980). Spine deficiency and polymorphism in a population of *Gasterosteus aculeatus*: an adaptation to predators?. Can. J. Zool.

[b59] Reimchen TE (1983). Structural relationships between spines and lateral plates in threespine stickleback (*Gasterosteus aculeatus*. Evolution.

[b61] Reimchen TE (1992). Injuries on stickleback from attacks by a toothed predator (*Oncorhynchus*) and implications for the evolution of lateral plates. Evolution.

[b62] Reimchen TE, Bell MA, Foster SA (1994). Predators and morphological evolution in threespine stickleback. The evolutionary biology of the threespine stickleback.

[b63] Reimchen TE (2000). Predator handling failures of lateral plate morphs in *Gasterosteus aculeatus*: implications for stasis and distribution of the ancestral plate condition. Behavior.

[b64] Schluter D (2001). Ecology and origin of species. Trends Ecol. Evol.

[b65] Schluter D, Marchinko KB, Barrett RDH, Rogers SM (2010). Natural selection and the genetics of adaptation in threespine stickleback. Philos. Trans. R. Soc. Lond. B Biol. Sci.

[b66] Shapiro MD, Marks ME, Peichel CL, Blackman BK, Nereng KS (2004). Genetic and developmental basis of evolutionary pelvic reduction in threespine sticklebacks. Nature.

[b67] Spence R, Wootton RJ, Przybylski M, Macdonald K, Smith C (2012). Calcium and salinity as selective factors in plate morph evolution of the three-spined stickleback (*Gasterosteus aculeatus*. J. Evol. Biol.

[b68] Taylor EB, McPhail JD (1986). Prolonged and burst swimming in anadromous and fresh-water threespine stickleback, *Gasterosteus aculeatus*. Can. J. Zool.

[b69] Thompson DW (1917). On growth and form.

[b70] Tulley JJ, Huntingford FA (1987). Parental care and the development of adaptive variation in anti-predator responses in sticklebacks. Anim. Behav.

[b71] Watt J (1995). Seasonal and area-related variations in the diet of otters *Lutra lutra* on Mull. J. Zool.

[b72] Withler RE, McPhail JD (1985). Genetic variability in freshwater and anadromous sticklebacks (*Gasterosteus aculeatus*) of southern British Columbia. Can. J. Zool.

[b73] Wootton RJ (1976). The biology of sticklebacks.

[b74] Wootton RJ (1984). A functional biology of sticklebacks.

[b75] Wootton RJ (2009). The Darwinian stickleback *Gasterosteus aculeatus*: a history of evolutionary studies. J. Fish Biol.

[b76] Zeller M, Lucek K, Haesler M, Seehausen O, Sivasundar A (2012). Little evidence for a selective advantage of armour-reduced threespined stickleback individuals in an invertebrate predation experiment. Evol. Ecol.

